# UV-Assisted Photochemical Synthesis of Reduced Graphene Oxide/ZnO Nanowires Composite for Photoresponse Enhancement in UV Photodetectors

**DOI:** 10.3390/nano8010026

**Published:** 2018-01-05

**Authors:** Changsong Chen, Peng Zhou, Na Wang, Yang Ma, Haisheng San

**Affiliations:** 1 Pen-Tung Sah Institute of Micro-Nano Science and Technology, Xiamen University, Xiamen 361005, China; chencs@stu.xmu.edu.cn (C.C.); zhoup@stu.xmu.edu.cn (P.Z.); wangn@stu.xmu.edu.cn (N.W.); mymymy@stu.xmu.edu.cn (Y.M.); 2 College of Electronic Science and Technology, Xiamen University, Xiamen 361005, China

**Keywords:** ZnO nanowire, ultraviolet (UV) photoresponse, reduced graphene oxide, UV-assisted photochemical synthesis

## Abstract

The weak photon absorption and high recombination rate of electron-hole pairs in disordered zinc oxide nanowires (ZNWs) limit its application in UV photodetection. This limitation can be overcome by introducing graphene sheets to the ZNWs. Herein we report a high-performance photodetector based on one-dimensional (1D) wide band-gap semiconductor disordered ZNWs composited with reduced graphene oxide (RGO) for ultraviolet (UV) photoresponse enhancement. The RGO/ZNWs composites have been successfully synthetized through UV-assisted photochemical reduction of GO in ZNWs suspension. The material characterizations in morphology, Raman scattering, and Ultraviolet-visible light absorption verified the formation of graphene sheets attached in ZNWs network and the enhancement of UV absorption due to the introduction of graphene. In comparison with photodetectors based on pure ZNWs, the photodetectors based on RGO/ZNWs composite exhibit enhanced photoresponse with photocurrent density of 5.87 mA·cm^−2^, on/off current ratio of 3.01 × 10^4^, and responsivity of 1.83 A·W^−1^ when a UV irradiation of 3.26 mW·cm^−2^ and 1.0 V bias were used. Theory analysis is also presented to get insight into the inherent mechanisms of separation and transportation of photo-excited carriers in RGO/ZNWs composite.

## 1. Introduction

Ultraviolet (UV) light detection is important for many applications such as environmental monitoring, flame detection, military applications, and industrial quality control [1,2,3,4,5]. The development of UV photodetectors using wide band gap materials has progressed considerably during the last two decades [6,7]. Therein, ZnO, as a wide direct band gap (3.37 eV) compound semiconductor with large exciton binding energy (60 meV), has been widely investigated for its application in UV photodetectors. ZnO has a rich family of nanostructures such as nanowires (NWs), nanobelts, nanoparticles, nanotips, and nanotubes [1,3,8]. One-dimensional (1D) ZnO nanowires (ZNWs) have attracted significant attention due to their large surface area, good crystal quality, and unique photonic properties. One particular advantage of ZNWs is that it is a low-cost material that can be solution-synthesized on a vast variety of substrates [5]. In the past few years, there has been an increasing interest in 1D ZNWs-based UV photodetectors and their potential applications in UV sensors, environmental monitors, and optical communications [4,9,10]. However, the UV photodetectors based on pure ZNWs suffer from the weak photosensitivity due to two main reasons: 1. the fast recombination of UV-excited electron-hole pairs (EHPs) in nanowires and 2. oxygen molecules (O_2_) adsorbed at the surface of ZNWs by capturing the electrons in the nanowires reduce the conductance of the nanowires. So far, many methods to enhance UV photoresponse of ZNWs have been investigated. The most effective way is to use the vertically oriented and crystallized ZNWs arrays to prepare the photodetectors. For example, Qin et al., reported an enhanced photoresponse of ZNWs [11]. It is suggested that the short and oriented nanowires can enhance the sensitivity of photoresponse due to its more direct and often faster pathway for electron transport. However, the oriented nanowire arrays integrated with cost-effective electrodes are considered more difficult to achieve for fast, low cost and large-scale device fabrication. In recent years the graphene, the 2-dimensional (2D) carbon nanomaterial, has attracted much attention in the application of ZNWs-based photodetectors owing to its good conductivity, superior chemical stability and high specific surface area [1,2,3,12,13,14,15,16]. Liao et al., reported a typical photodetectors based on ZnO single-NW sandwiched between two graphene sheets [9]. While the photoelectric performance was significantly improved compared to the conventional ZNWs-based photodetectors, ZnO single-NW UV sensors have very low photoresponse current due to the small size of individual nanowire, and high-precision measurement systems were also necessary to detect the weak signal, making the cost of such sensors prohibitive. Zhang et.al presented a high responsivity of UV photodetector using ZNWs arrays with a top transparent electrode made of a few-layer graphene sheets [5]. However, the photodetectors did not take full use of the merit of 1D nanowires with high specific surface area because the graphene sheets only contact nanowires in the nanowire ends. It has been well established that the Schottky barrier exists at the interface between ZNWs and graphene, which can effectively separate the EHPs generated by the UV irradiation. This will reduce the recombination of EHPs and result in the increase of photoresponse. Compared to the parallel oriented ZNW arrays, the disordered ZNWs can be easily covered by graphene sheets along the nanowire surface through a UV-assisted photochemical reduction of graphene oxide (GO) in ZNWs suspension [17]. This means that the disordered ZNWs combined with graphene sheets can be easily fabricated and assembled to the substrates with interdigitated electrodes (IDEs) in large-scale and low-cost preparation. So far, there are very few publications that studied the photoelectric performance of UV photodetectors using the disordered ZNWs/graphene composite.

In this work, we present a high-performance photodetector based on reduced graphene oxide (RGO)/ZNWs composite integrated on ceramics substrate with Au IDEs. The RGO/ZNWs composites have been successfully fabricated by UV-assisted photocatalytic reduction of GO by ZNWs in ethanol. In comparison with pure ZNWs-based devices, the RGO/ZNWs based photodetectors exhibited enhanced UV photoresponse.

## 2. Results and Discussion

**Figure 1:**
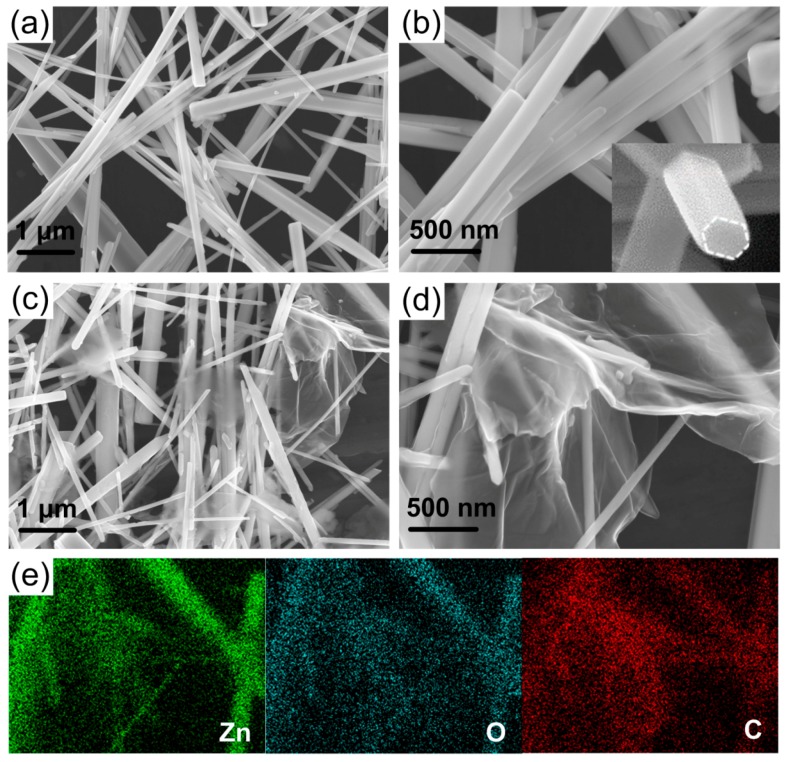
(**a**) Top-view and (**b**) partial enlarged field emission scanning electron microscopy (FESEM) images of one-dimensional (1D) ZnO nanowires (ZNWs), and the inset is the high magnification image of ZNWs; (**c**) Top-view and (**d**) partial enlarged FESEM images of reduced graphene oxide/ZNWs (RGO/ZNWs) composites with 1.0 wt % of RGO loaded; (**e**) Corresponding energy disperse spectrum (EDS) mappings of Zn, O, and C in RGO/ZNWs composites with 1.0 wt % of RGO loaded.

Figure 1a,b show top-view and partially enlarged field emission scanning electron microscopy (FESEM) images of 1D ZNWs coated on substrate, respectively. It is seen that the super-long and interlaced ZNWs chaotically stack in the substrate, and the majority of the nanowires are 45~200 nm in diameter and 5~50 μm in length. The high magnification image (see inset of Figure 1b) shows that the prepared ZNWs have long and straight walls with the crystalled hexagonal morphology. It has been well established that the length-diameter (aspect) ratio of ZNWs has significant influence on the performance of the photodetectors. The low aspect ratio was not beneficial to the full absorbance of UV radiation due to the relatively small surface area. In contrast, the high aspect ratio would make nanowires easily overlap each other, forming nanowire networks with electrically conductive pathways extended in all directions for carrier transport. The morphologies of ZNWs composited with 1.0 wt % of RGO are shown in Figure 1c,d. It can be seen that 2D RGO nanosheets, covering and tightly contacting with ZNWs, form a 3D graphene network, which also can be seen in RGO/ZNWs composites with 0.1, 0.5 and 8.0 wt % RGO contents (see Figure S1). To further identify the RGO nanosheets assembled to ZNWs, energy disperse spectrum (EDS) analysis were performed as shown in Figure 1e. The elemental mappings of C clearly indicate that RGO were uniformly distributed throughout these ZNWs.

**Figure 2:**
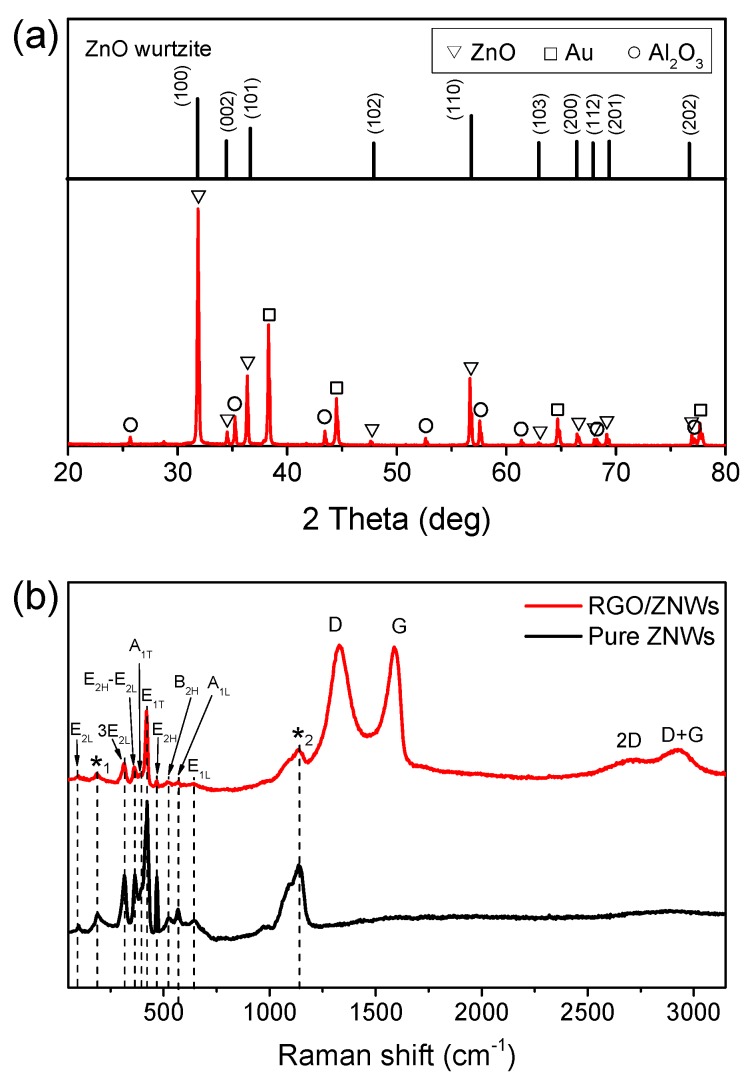
(**a**) X-ray diffraction (XRD) spectrum of ZNWs and (**b**) Raman spectra of pure ZNWs and RGO/ZNWs composite with 1.0 wt % of RGO loaded.

Figure 2a shows the X-ray diffraction (XRD) spectrum of the ZNWs. All of the diffraction peaks match the standard data for hexagonal wurtzite ZnO structure (JPCDS 36-1451) [18], which is well known to have highly semiconductive properties useful for photoelectrical applications. Raman spectroscopy is used to evaluate the quality and layer number of graphene layers. Figure 2b shows the comparison of Raman spectra of the pure ZNWs and the RGO/ZNWs composite with 1.0 wt % RGO loaded. Raman frequencies of the peaks are extracted by Lorentzian fits, and all the peaks are assigned according to Refs. [19,20,21,22] and listed in Table S1. As shown in Figure 2a, the ZNWs possess typical uniaxial wurtzite structure with fair crystal quality [23]. Moreover, the peaks at ~203 cm^−1^ (*_1_) and ~1145 cm^−1^ (*_2_) should belong the overtone process of acoustic phonon with A_1_ symmetry and the multiphonons process, respectively [19,24]. Four prominent graphene peaks at ~1327, ~1587, ~2650 and ~2926 cm^−1^, corresponding to D, G, 2D and D + G band respectively, are observed in the Raman spectra of RGO/ZNWs composite. The prominent D band and G band show typical features of RGO [25,26]. At the same time, the sharp G band implies that a significant amount of sp^2^ carbon networks were generated during photocatalytic reduction [26,27]. Furthermore, the weak and broadened 2D and D + G peak indicates the generation of multi-layered graphene with a few defects [28]. The multiple layers of RGO nanosheets that overlapped and interconnected each other to form a graphene network which ensures a conductive pathway when it will contact with the ZNWs.

**Figure 3:**
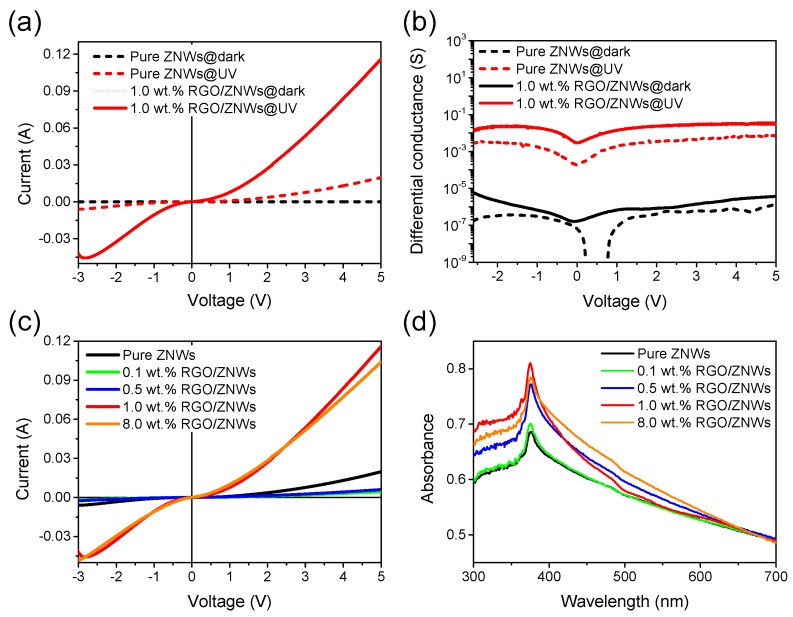
(**a**) *I-V* curves and (**b**) differential conductance curves of photodetectors based on pure ZNWs and 1.0 wt % RGO/ZNWs composite in dark and under ultraviolet (UV) irradiation with power density of 2.90 mW·cm^−2^; (**c**) *I*-*V* curves of photodetectors based on pure ZNWs and different RGO contents of RGO/ZNWs composites under UV irradiation with power density of 2.90 mW·cm^−2^; (**d**) Ultraviolet-visible light (UV-VIS) absorption spectra of pure ZNWs and different RGO contents of RGO/ZNWs composites.

Figure 3a shows a comparison of *I*-*V* curves of photodetectors based on pure ZNWs and 1.0 wt % RGO/ZNWs composite in dark and under UV irradiation. It can be seen that the photodetectors in dark did not exhibit any appreciable change in *I*-*V* curves whether or not there is RGO composited with ZNWs. Under UV irradiation, however, the pure ZNWs device exhibited a light increase in curve slope (conductance), and a more significant change in curve slope occurred in the photodetector based on 1.0 wt % RGO/ZNWs composite. Meanwhile, the symmetric *I*-*V* curves about 0 V point also indicate that there are two back-to-back Schottky contacts on the devices, and both curves exhibit a nonlinear characteristic due to the metal/semiconductor/metal structure. In order to get insight into the effect of the graphene on the conductance of composites, the typical differential conductance spectra are presented in Figure 3b. It is seen that the d*I*/d*V* spectra of photodetectors based on pure ZNWs and 1.0 wt % RGO/ZNWs composite have a little difference when devices are in the dark, but in a very low conductance level. This implies that the RGO have weak influence on the conductance of ZNWs in dark. Under UV irradiation, the d*I*/d*V* values of pure ZNWs-based and 1.0 wt % RGO/ZNWs-based devices are increased to a high level that are around 3 to 4 orders of magnitude higher than that in dark, while the 1.0 wt % RGO/ZNWs-based device is about an order of magnitude higher than pure ZNWs-based device. It can be well understood that the EHPs excited by the UV incident light with energy larger than the band gap of ZnO increase the free carrier concentration, and thus results in the increase of conductivity of ZNWs. Furthermore, as the RGO were composited to the ZNWs, the graphene nanosheets were attached to the surface of ZNWs and filled into the gap of nanowires, facilitating the formation of conductive graphene network. The graphene network would be beneficial to the charge collection and transport due to not only its high conductivity but also its excellent electrical contact with NWs. The graphene sheets are capable of rapidly collecting the electrons from ZNWs and thus effectively suppress their recombination with holes in ZNWs (will be discussed in work mechanisms part). As a result, the conductivity of RGO/ZNWs is significantly increased.

In order to determine the effect of RGO content on the enhancement of UV photoresponse, different content of RGO were composited with ZNWs in combination with the photoresponse measurements for assessing the optimal content of RGO in composites. As shown in Figure 3c, a comparison of *I-V* curves of photodetectors based on the pure ZNWs and the RGO/ZNWs composite prepared using different contents of RGO, such as 0.1, 0.5, 1.0 and 8.0 wt %. Under UV irradiation with power density of 2.90 mW·cm^−2^, it can be seen that the *I*-*V* curve of photodetector with 1.0 wt % RGO/ZNWs composite has the maximum conductance, and the conductance cannot be further increased when the content of graphene is continually increased up to 8%, which implies that the 1.0 wt % RGO/ZNWs composite has the optimization. Meanwhile, it is noted that the devices based on composite with 0.1 and 0.5 wt % RGO generated a lower photo-excited current in comparison with pure ZNWs at same bias-voltage. To understand the change in photoresponse due to the introduction of RGO in ZNWs, we investigated the effect of the RGO on the UV absorption of ZNWs. Figure 3d shows the ultraviolet-visible light (UV-VIS) absorption spectra of the ZNWs and the RGO/ZNWs composites prepared using different contents of RGO. It is found that the pure ZNWs and all of RGO/ZNWs composites show a strong absorption peak at 375 nm, and the RGO/ZNWs composites exhibit an enhanced absorption over a broad region ranging from 300 to 600 nm except for the 0.1 wt % RGO/ZNWs composite due to the low amount of RGO in this composite. These results are similar to those reported in the combination of ZnO and graphite-like carbon layers or RGO [17,29,30]. Such an enhanced absorption in the visible light region is due to the absorption contribution from RGO, while an enhanced absorption in UV-region with the increase of RGO content in the RGO/ZNWs composite is attributed to the increase of surface electric charge of ZnO due to the introduction of graphene in the composites, which may modify excitation formation upon irradiation [29]. Since the photogenerated current is related with the generation and recombination of EHPs, the significant EHPs excited due to enhanced UV absorption and their effective separation can be used to explain the change in photoelectric performance. It is noticeable that the 1.0 wt %. RGO/ZNWs composite exhibits a maximum absorption in UV region, directly accounting for the maximum slope in *I*-*V* curve of 1.0 wt % RGO/ZNWs-based device. With the higher graphene loading (e.g., 8.0 wt % RGO content), the UV absorbance shows some reduction instead of being further improved. It is suggested that excessive graphene increases the light scattering and leads to the reduction of ZnO adsorption because the ZnO is surrounded by graphene. While the composites containing low amounts of RGO (e.g., the 0.1 wt % and 0.5 wt % RGO composite) did not exhibit less absorption than pure ZnO, they generated a lower photocurrent than the pure ZnO at the same bias-voltages. This could be attributed to the formation of discontinuous graphene islands anchored in ZNWs due to the lack of graphene, which are seen as a kind of recombination center instead of a continuous electron pathway [31].

**Figure 4:**
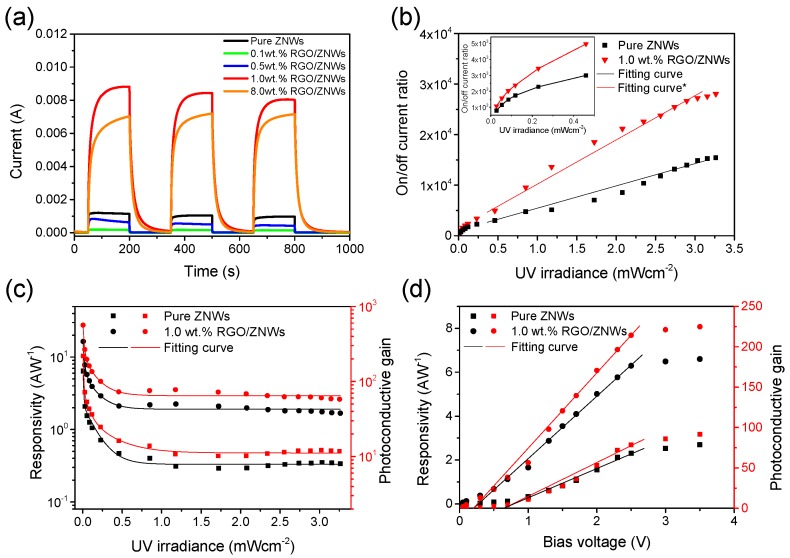
(**a**) Time-dependent photoresponses of photodetectors based on ZNWs and RGO/ZNWs composited with different contents under 3.26 mW·cm^−2^ of UV irradiation controlled by switching on/off cycles and at a bias-voltage of 1.0 V; (**b**) Calculated on/off current ratio of photodetectors based on ZNWs and 1.0 wt % RGO/ZNWs composite. The inset is the magnification of on/off current ratio in low irradiation intensity; (**c**) UV intensity dependence of photoconductive gain and responsivity of photodetectors based on ZNWs and 1.0 wt % RGO/ZNWs composite at bias-voltage of 1.0 V; (**d**) Bias-voltage dependence of photoconductive gain and responsivity of photodetectors based on ZNWs and 1.0 wt % RGO/ZNWs.

Photoresponse experiments were performed to investigate the photoelectric interaction between ZNWs and graphene. Figure 4a shows the time-dependent photoresponses of photodetectors based on ZNWs and different contents of RGO/ZNWs composite under 3.26 mW·cm^−2^ of UV irradiation controlled by switching on/off cycles and at a bias-voltage of 1.0 V. It is clear that uniform and repeatable photocurrent responses were observed for each switch-on and switch-off, and photodetector based on 1.0 wt % RGO/ZNWs composite generated maximum photocurrent density of 5.87 mA·cm^−2^ in the maximum power density of UV irradiation (3.26 mW·cm^−2^). Meanwhile, it is found that the rise-time *t*_r_ and fall-time *t*_d_ of ZNWs-based device were 2.42 and 0.20 s, respectively, which are faster than those of RGO/ZNWs based device (*t*_r_ = 28.46 s and *t*_d_ = 9.76 s). It has been well established that the EHP generation and the oxygen chemisorption dominate the rise process and fall process of photoresponse of ZnO UV detectors, respectively [32]. In RGO/ZNWs composites, the ZNWs were covered by RGO nanosheets, which means that the oxygen in atmosphere cannot directly and fully contact with the ZnO surface. This would suppress the absorption and desorption of oxygen in the surface of ZNWs, resulting in a slower response time than that of ZNWs-based photodetector [33]. The on/off current ratio is defined as *∆I*_d_/*I*_d_, where *∆I*_d_ = *I*_ph_ − *I*_d_ and *I*_ph_ and *I*_d_ are the current when the UV irradiation is switched on and off, respectively. By changing the power density of UV irradiation, we measured the one on/off cycle of photoresponses of photodetectors based on ZNWs and 1.0 wt % RGO/ZNWs composite (see Figure S2a), and calculated their on/off current ratios (*∆I*_d_/*I*_d_). As shown in Figure 4b, the on/off current ratio of photodetector based on 1.0 wt % RGO/ZNWs composite was measured to be 30,102 when the UV power density reaches 3.26 mW·cm^−2^. This value is around 3 times as high as that of pure ZNWs-based photodetector (∼9900), which indicates that the RGO composited into ZNWs is beneficial to the increase of photoresponse performance of photodetector. Furthermore, the photocurrent on/off ratios of both types of photodetectors first increases exponentially in low power region (see the inset of Figure 4b), and then increase linearly with the increase of UV power, and finally reaches a saturation value (about 2.8 × 10^4^ for RGO/ZNWs and 1.5 × 10^4^ for pure ZNWs) when the UV power density exceed 3.0 mW/cm^2^. From the inset of Figure 4b, it is found that a minimum on/off current ratio of 5.14 × 10^2^ (or the photocurrent density of 0.10 mA·cm^−2^) were still observed when the lowest power density of the UV lamp (∼6 μW·cm^−2^) was used. Meanwhile, the linear ∆*I*_d_/*I*_d_ at medium UV irradiation is consistent with other works [5,34], which is due to that the concentration of the photogenerated EHPs is proportional to the absorbed flux. A gradual saturation of ∆*I*_d_/*I*_d_ observed at higher UV irradiances can be explained by the flattening of the band bending and narrowing of the surface depletion layer at high irradiation intensity (will be discussed in work mechanisms part), resulting in a dynamic balance between generation and recombination of photo-excited EHPs.

The photoelectric characteristics are also evaluated by another two parameters, namely responsivity (*R*_s_) and photoconductive gain (*G*). The *R*_s_ is defined as *R*_s_ = *I*_ph_/*P*_opt_, where *P*_opt_ is the effective light power absorbed in active region of devices. *G* is defined as the number of electrons collected by electrodes due to the excitation by one incident photon, which can be expressed as *G* = (*I*_ph_/*P*_opt_) × (*hv*/*e*), where *hν* is the energy of the incident photon, and *e* is the electron charge.

Figure 4c displays the incident power density dependence of the *G* and *R*_s_ curves of ZNWs-based and 1.0 wt % RGO/ZNWs-based photodetectors at a bias-voltage of 1.0 V. It can be seen that both *R*_s_ and *G* curves rapidly decrease at low incident power and then tend to a stable value. The 1.0 wt % RGO/ZNWs-based photodetector exhibits a stable responsivity of ~2.0 A·W^−1^ and an average photoconductive gain of 71.97 under a wide range of UV irradiation (0.5~3.26 mW·cm^−2^), which is more than ~6 times as high as those of pure ZNWs-based photodetector. This behavior is related to the photoconduction mechanism of the nanowires and can be explained by the flattening of the band bending and narrowing of the surface depletion layer [5]. Another key parameter to affect the photoelectric performance is the bias voltage (*V*_b_) applied in the IDEs. We measured one on/off cycle of photoresponses of pure ZNWs-based and 1.0 wt % RGO/ZNWs-based photodetectors under 3.26 mW·cm^−2^ of UV irradiation and at different *V*_b_ (see Figure S2b), and calculated their *R_s_* and *G* values at different *V*_b_, as shown in Figure 4d. It can be seen that *R*_s_ and *G* curves of two types of photodetectors exhibit a positive linear relation with *V*_b_ from 0.2 to 2.5 V, and both curves tend to saturation when the bias exceed 2.5 V where *R*_s_ and *G* values of photodetectors with RGO are about 2 times as high as that without RGO. Such a linear relation would imply that the variation of carrier mobility in materials is related to *V*_b_ and is in a linear range [35]. Since the photo-excited electrons are transferred to graphene layers and rapidly transport in the graphene network, the mobility of electrons in RGO/ZNWs composite will be far higher than that in pure ZNWs. This can be verified by the slope of the straight *G* curve of photodetector with RGO (*G*/*V*_b_ = 95.5 V^−1^) that is larger than that of photodetector without RGO (*G*/*V*_b_ = 38.1 V^−1^). It means that electrons in RGO/ZNWs composites will take shorter time to be rapidly transferred to electrodes than pure ZNWs, and thus a lower carrier recombination opportunity. As the *V*_b_ is enough high and exceed a critical value, the *G* or *R*_s_ will reach a saturation due to the cancellation of carrier recombination.

**Figure 5:**
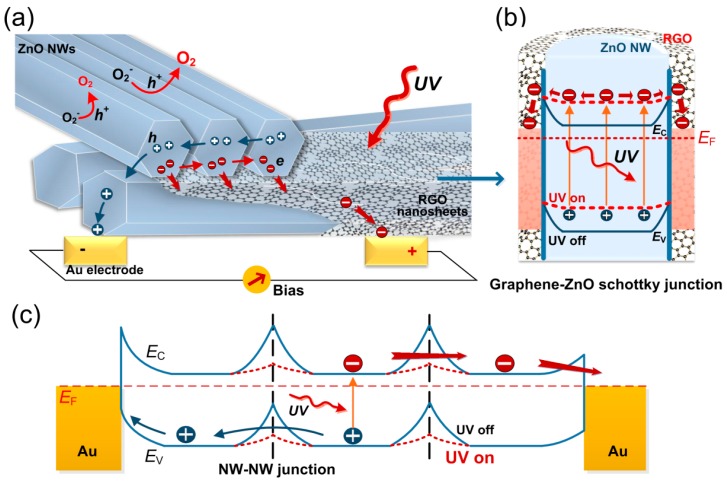
Schematic illustrations of work mechanisms of photodetectors based on RGO/ZNWs composite. (**a**) Charge transfer in ZNWs/ZNWs, RGO/ZNWs, and ZNWs (or RGO)/electrodes; Energy band diagrams of the contact interfaces in (**b**) RGO/ZNW and (**c**) ZNWs/ZNWs and their modifications in the profiles of conduction band and valence band along the nanowire radius and length in the dark and under UV irradiation.

Based on the above photoelectric measurement results as shown in Figure 4, we can get insight into the inherent mechanisms of separation and transportation of photo-excited EHPs in the photodetectors based on pure ZNWs and RGO/ZNWs composite. As shown in Figure 5, two kinds of contact interfaces, namely ZNWs/ZNWs and RGO/ZNWs, are presented in the same transducing structure and are schematiclly illustrated through the energy band diagrams. When compared to ZNWs-based devices, the RGO/ZNWs-based photodetector provides multiple strategies in photoresponse enhancement by suppressing carrier recombination and improving carrier transport. Firstly, the combination of 2D graphene sheets and 1D ZNWs creates an extremely large interface area of graphene/ZnO due to the high specific surface area of 1D and 2D nanomaterials. Therefore, graphene/ZNWs based structure has more significant transport efficency of carriers than ZNWs-based structure. Secondly, the graphene sheets covered in the surface of ZNWs can effectively suppress carrier recombination. Schottky junction can be formed in the interface of graphene/ZnO since the work function of ZnO (4.45 eV) is less than that of graphene (4.70 eV), by which the photo-excited EHPs can be effectively separated along the interface of graphene/ZnO in overall length (see Figure 5a). This means that EHPs recombination will be greatly reduced. As a result, the free carrier lifetime is increased and thus the photoresponse is enhanced. Thirdly, the graphene sheets making a fully electrical contact with ZNWs are capable of rapidly collecting the electrons from ZnO and transporting electrons to electrodes. Based on the high-efficent EHPs separation, the increased carrier density in ZnO by the UV irradiation reduces the work function of ZnO, resulting in the flattening of the band bending and narrowing of the surface depletion layer (see Figure 5b). This will facilitate the electrons in the conduction band to readily travel across the interface and transfer into the graphene, while keeping the holes in valence band to transport in ZNWs. With an external electric field, the electrons transport from graphene sheets to the Au electrodes while the holes transport through the interior of ZNWs. The separated transport pathways for the electrons and holes also significantly decrease the recombination of electrons and holes (see Figure 5a) and enhance the photoresponse current of photodetectors. In contrast, the energy band of ZNWs-based structure is dominated by the junction barriers between neighboring wires (NW-NW junction barriers) [36,37]. Similarly, with an UV irradiation the carrier density in NWs will increase, which will narrow the barrier width and lower the effective barrier height beneficial to the carrier transportation (see Figure 5c). Nevertheless, the transport efficiency of carriers in ZNWs is far less than that in graphene/ZNWs. This is due to the fact that NWs-NWs contacts have very small point-contact area and intricate transport pathway, resulting in the increase in transport time and resistance, and thus the increase of carrier recombination. Furthermore, oxygen molecules will be adsorbed onto the exposed surface of ZNWs by capturing free electrons from the ZnO [O_2_(g) + e^−^ → O_2_^−^(ad)], resulting in the reduction of ZnO conductance.

## 3. Materials and Methods

### 3.1. Fabrication of Ultraviolet (UV) Photodetectors Based on Reduced Graphene Oxide/ZnO Nanowires (RGO/ZNWs) Composite

**Figure 6:**
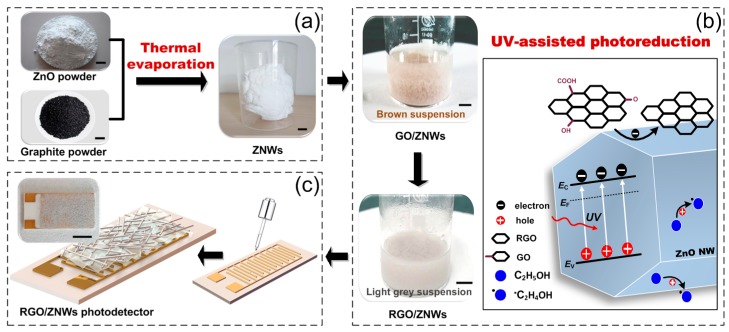
Schematic illustration of fabrication processes of UV photodetectors based on RGO/ZNWs composites. (**a**) 1D ZNWs prepared using a mixture of ZnO and graphite powders through thermal evaporation; (**b**) RGO/ZNWs composite prepared using UV-assisted photochemical reduction of GO in ZNWs suspension; (**c**) RGO/ZNWs based photodetector prepared by adding RGO/ZNWs solutions to the surface of substrates with IDEs. The scale bars represent 5 mm.

1D ZNWs were prepared using a homogeneous mixture of commercial ZnO and graphite powder at a proportion of 1:3 as the source by a simple thermal evaporation in a tube furnace in a mixed atmosphere of oxygen and inert gas, as shown in Figure 6a. The temperature was controlled to 800–1250 °C for 10–60 min to form crystallized wurtzite phase. Fine graphite powder was used to promote growth of ZNWs, and it was easily removed by oxidation in flowing O_2_ at 700 °C for 1–3 h. Before the reduction of GO, 10 mg commercial GO powder was exfoliated in 10 mL ethanol by ultrasonication for 120 min to form a brown GO colloidal dispersion. Next, 1.0 g ZnO nanowires were immersed into above GO dispersion by ultrasonication and magnetic stirring for 30 min to produce light-brown uniform dispersion, as shown in Figure 6b. In the GO/ZNWs solution, the functional groups on GO could form robust bonding with ZnO, which would facilitate the following UV-assisted reduction of GO. UV-assisted reduction of GO by ZNWs in ethanol was carried out in a 25 mL transparent reaction glass vessel and the mixed solution was exposed to UV irradiation given by a 500 W high pressure Hg lamp (MERC-JY500G, Beijing, China) at a main wavelength of 365 nm with magnetic stirring for 2 h. After UV irradiation, it was obviously found that the color of suspension changed into light gray, as shown in Figure 6b, which is as a result of the successful chemical reduction of GO sheets. In the present of ethanol, the electrons generated by ultraviolet photons transfer from the excited ZNWs to the GO to produce RGO as following reactions:

(1) $$\mathrm{ZnO}+hv\to \mathrm{ZnO}(h+e)\overset{C_{2}H_{5}\mathrm{OH}}{\to }\mathrm{ZnO}(e)+{}^{•}C_{2}H_{4}\mathrm{OH}+H^{+}$$

(2) ZnO(e)+GO→ZnO+RGO

With an excitation of UV light, the EPHs are produced in ZNWs, the holes are scavenged to produce ethoxy radicals and thus leave the electrons within ZNWs (reaction (1)). The accumulated electrons serve to interact with the GO nanosheets in order to reduce certain functional groups [38].

**Figure 7:**
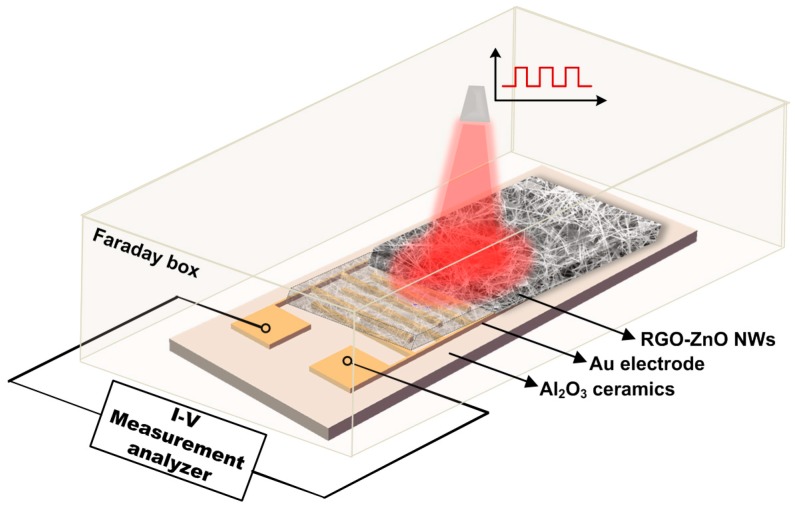
Schematic structure of RGO/ZNWs-based UV photodetectors and its measurement system.

Figure 7 shows the schematic structure of RGO/ZNWs based UV photodetectors. Au IDEs were fabricated on 2.0 × 1.0 cm^2^ of aluminum oxide (Al_2_O_3_) ceramics substrates by standard photolithography and lift-off process (see Figure S3). The optical active area is 1.5 × 1.0 cm^2^ containing 37 pairs of Au electrode fingers with finger-width of 0.1 mm, finger-thickness of 2.5 μm, and finger-spacing of 0.1 mm. As shown in Figure 6c, the as-synthesized RGO/ZNWs solutions were added to the surface of substrates with IDEs, followed by air drying. This process was repeated for 5 times to form about 30 µm thick RGO/ZNWs multiple layers on the surface of IDEs. To optimize the photoelectric performance of the composites, the component proportion of RGO/ZNWs solution was performed in a controlled experimental investigation. The composite solutions with different RGO contents of 0.1, 0.5, 1.0, 8.0 wt % were synthesized to prepare different test samples (see Figure S4) according to above same preparation processes.

### 3.2. Materials Characterizations and Device Measurements

Morphologies and size analysis of the ZNWs and RGO/ZNWs were characterized using FESEM (ZEISS microscope, Oberkochen, Germany). The EDS analysis was carried out using energy dispersive spectroscopy (ZEISS SUPRA55, Oberkochen, Germany). The crystal structures of the samples were characterized by XRD analysis (Rigaku Ultima IV, Tokyo, Japan). Raman scattering studies were performed by a Raman spectrometer (Renishaw in via, London, UK) using laser wavelength of 532 nm, which provides valuable information about the defects and stacking of graphene sheets. UV-VIS absorption spectra of the sample filtrates were measured by UV-VIS spectrophotometer (Cary 300, Santa Clara, CA, USA).

The photoresponse properties of the photordetectors were characterized using an electrochemical workstation (Chenhua CHI660E, Shanghai, China) under dark and room temperature in a Faraday cage. The photodetectors were clamped on the probe station with two standard probes mechanically pressing on electrode pads of the photodetectors. In measurement, a bias-voltage or scanning voltage was applied to the device, and the data were recorded by the workstation. A 3.08 W UV lamp (NICHIA NCSU033B, Tokushima, Japan) was used as the UV irradiation source with tunable irradiation power intensity controlled by the direct current power supply (Agilent E3631A, Santa Clara, CA, USA).

## 4. Conclusions

We fabricated a high-performance photodetector based on 1D wide band-gap semiconductor disordered ZNWs composited with RGO for UV photoresponse enhancement. RGO/ZNWs composite were successfully synthetized through UV-assisted photochemical reduction of GO in ZNWs suspension. The photodetectors can be prepared in large-scale for practical applications by coating the RGO/ZNWs composite on ceramics substrates with Au IDEs. The material characterizations in morphology and Raman scattering indicate the generation of multiple layers of RGO nanosheets in the surface and gap of ZNWs, which provide a conductive pathway due to the formation of conductive graphene network. The introduction of 1.0 wt % RGO in ZNWs can realize the maximum UV absorption, which was verified by the measurements in Ultraviolet-visible light absorption and conductivity measurements. The photodetectors based on RGO/ZNWs composite exhibited enhanced photoresponse performance with photocurrent density of 5.87 mA·cm^−2^, on/off current ratio of 3.01 × 10^4^, photoconductive gain of 61.8, and responsivity of 1.83 AW^−1^, when a UV irradiation of 3.26 mW·cm^−2^ and 1.0 V bias were used. Theory analysis indicates that the RGO/ZNWs-based photodetector provides multiple strategies in photoresponse enhancement by the extremely large interface area of graphene/ZNWs to suppress carrier recombination and improve carrier transport.

## Supplementary Materials

The following are available online at http://www.mdpi.com/2079-4991/8/1/26/s1, Figure S1: SEM images of RGO/ZNWs composites with (a) 0.1 wt %; (b) 0.5 wt % and (c) 8.0 wt % RGO contents. Table S1: Summarization of the Raman frequencies of 1.0 wt % RGO/ZNWs composites sample with assignments of all the peaks, Figure S2: (a) Time-dependent UV photocurrents of 1.0 wt % RGO/ZNWs-based photodetector at 1.0 V bias-voltage and under various UV irradiations with one on/off cycle. (b) Time-dependent UV Photocurrents of 1.0 wt % RGO/ZNWs-based photodetector at various bias-voltages and under 3.26 mW·cm^−2^ of UV illumination with one on/off cycle. Figure S3: Fabrication Illustration of the Au interdigitated electrodes on Al_2_O_3_ ceramics substrates, Figure S4: (a) Photographs of (a) interdigitated Au electrode substrate, (b) ZNWs-based photodetector and RGO/ZNWs-based photodetectors with (c) 0.1 wt %, (d) 0.5 wt %, (e) 1.0 wt % and (f) 8.0 wt % RGO loaded.
