# Supplementary material for: UV-Assisted Photochemical Synthesis of Reduced Graphene Oxide/ZnO Nanowires Composite for Photoresponse Enhancement in UV Photodetectors
Source: Nanomaterials (Basel). 2018 Jan 5;8(1):26. doi: 10.3390/nano8010026 (PMC5791113; doi:10.3390/nano8010026)
Supplement: Supplementary file 1 [file nanomaterials-08-00026-s001.pdf]

Supporting Information

Article

UV-Assisted Photochemical Synthesis of Reduced Graphene Oxide/ZnO Nanowires Composite for Photoresponse Enhancement in UV Photodetectors

Changsong Chen [1], Peng Zhou [1], Na Wang [1], Yang Ma [1] and Haisheng San [1,2,*]

[1] Pen-Tung Sah Institute of Micro-Nano Science and Technology, Xiamen University, Xiamen 361005, China; chencs@stu.xmu.edu.cn (C.C.); zhoup@stu.xmu.edu.cn (P.Z.); wangn@stu.xmu.edu.cn (N.W.); mymymy@stu.xmu.edu.cn (Y.M.)

[2] College of Electronic Science and Technology, Xiamen University, Xiamen 361005, China

* Correspondence: sanhs@xmu.edu.cn; Tel.: +86-592-218-1340

1. SEM images of RGO/ZNWs composites with various RGO

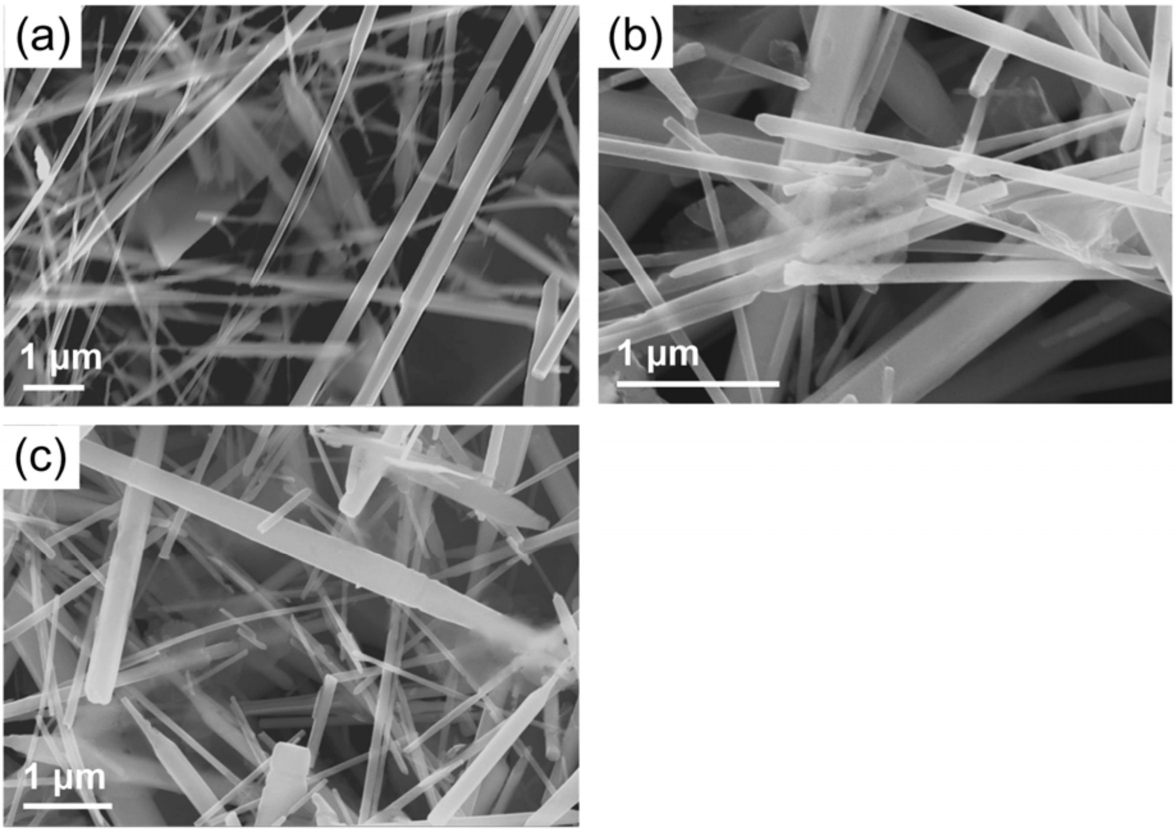

Figure S1. SEM images of RGO/ZNWs composites with (a) 0.1 wt %; (b) 0.5 wt % and (c) 8.0 wt % RGO contents.

2. Assignments of Raman peaks of RGO/ZNWs composite

Table S1. Summarization of the Raman frequencies of 1.0 wt.% RGO/ZNWs composites sample with assignments of all the peaks.

Peak	1	2	3	4	5	6	7	8	9	10	11	12	13	14	15
ω_{RGO}(cm^{-1})												1327	1587	2704	2926
ω_{ZnO}(cm^{-1})	98	203	315	362	391	420	460	522	570	638	1145				
Assignment	E_{2L}	$*_1$	$3E_{2L}$	E_{2H}-E_{2L}	A_{1T}	E_{1T}	E_{2H}	B_{2H}	A_{1L}	E_{1L}	$*_2$	D	G	2D	D+G

3. Photoresponse currents of photodetectors based on RGO/ZNWs composite

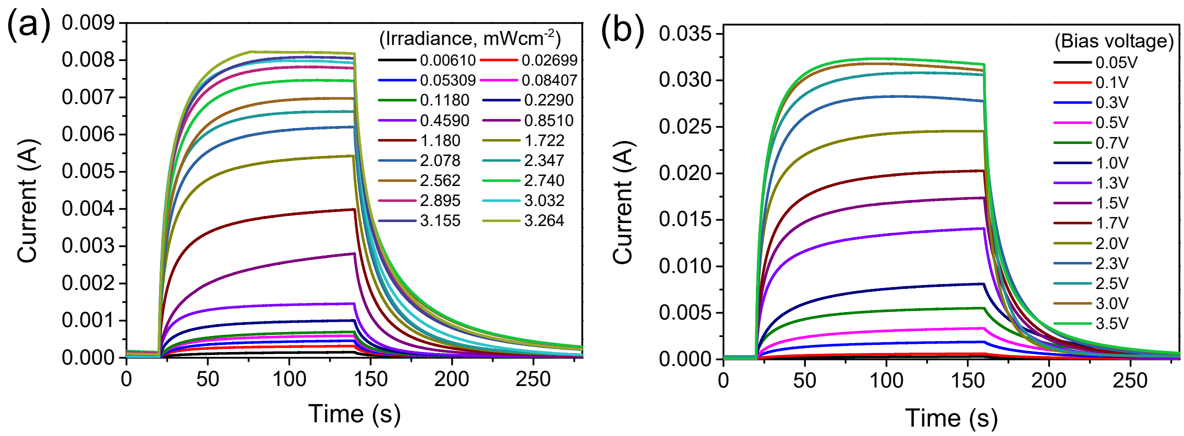

Figure S2. (**a**) Time-dependent UV photocurrents of 1.0 wt % RGO/ZNWs-based photodetector at 1.0 V bias-voltage and under various UV irradiations with one on/off cycle; (**b**) Time-dependent UV Photocurrents of 1.0 wt % RGO/ZNWs-based photodetector at various bias-voltages and under 3.26 mW·cm^{-2} of UV illumination with one on/off cycle.

4. Fabrication of Au interdigitated electrodes

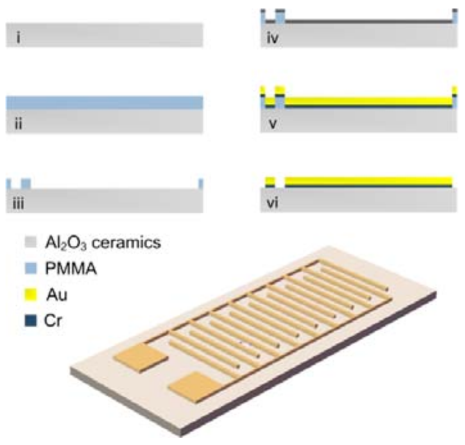

Figure S3. Fabrication Illustration of the Au interdigitated electrodes on Al_2O_3 ceramics substrates.

Thermal evaporation and standard photolithography process were employed for depositing the Au interdigitated electrodes (IDEs) on Al2O3 ceramics substrates (20 mm × 10 mm × 0.635 mm). Firstly, Al2O3 ceramics substrates were degreased and cleaned using acetone, isopropanol, and deionized water in ultrasonic bath for 15 min, respectively (see Figure S1(i)), and then a photoresist (polymethylmethacrylate, PMMA) were coated evenly in the ceramics substrates (see Figure S1(ii)). Next, a negative image of the desired electrode patterns was shaped in the photoresist using photolithographic technology, following by a photoresist development process (see Figure S1(iii)). Following the above step, the Cr (100 nm)/Au (2.5 μm) films were deposited on the mask substrates using the magnetron sputtering and electron-beam evaporation methods (see Figure S1(iv) and (v)). Finally, the lift-off process was used to remove mask (see Figure S1(vi)).

5. Photographs of photodetectors based on ZNWs and RGO/ZNWs

Photographs of the fabricated interdigitated Au electrodes on Al2O3 ceramics substrate and UV photodetectors with different contents of RGO loaded are shown in Figure S2. 37 pairs of Au electrodes with uniform finger width and spacing width were deposited on ceramics substrate. It can be seen that ZNWs or RGO/ZNWs composite were coated evenly on substrate, with colors varying from pure white to light grey as the increase in RGO content.

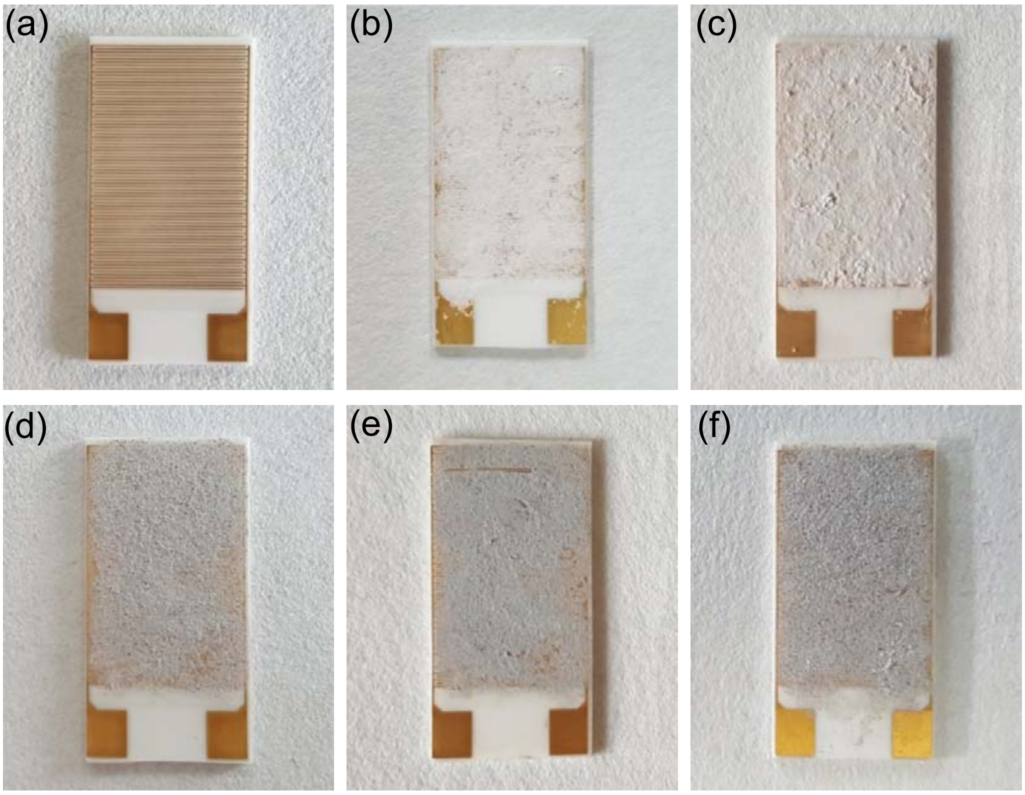

Figure S4. (a) Photographs of (a) interdigitated Au electrode substrate, (b) ZNWs-based photodetector and RGO/ZNWs-based photodetectors with (c) 0.1 wt %, (d) 0.5 wt %, (e) 1.0 wt % and (f) 8.0 wt % RGO loaded.
